# Shining Light on Inverted Singlet–Triplet Emitters

**DOI:** 10.1021/acs.jctc.3c01112

**Published:** 2023-11-22

**Authors:** Matteo Bedogni, Davide Giavazzi, Francesco Di Maiolo, Anna Painelli

**Affiliations:** Department of Chemistry, Life Science and Environmental Sustainability, Università di Parma, 43124 Parma, Italy

## Abstract

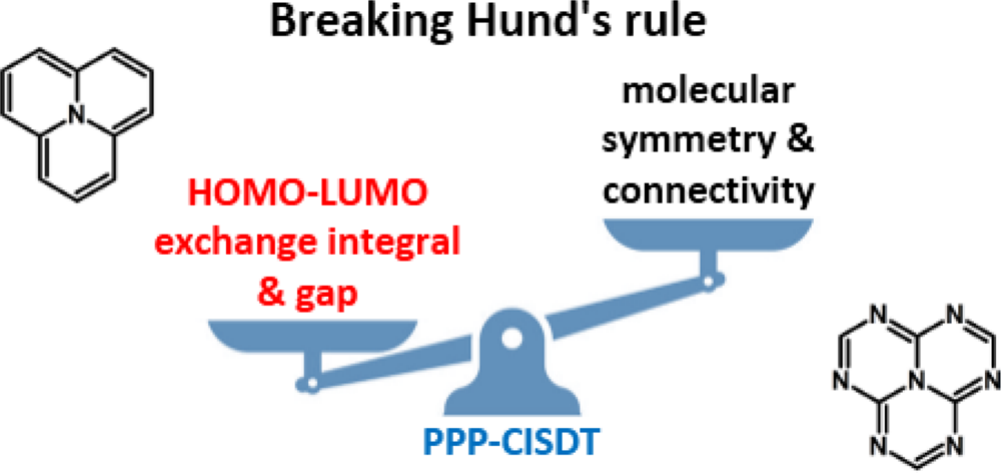

The inversion of the lowest singlet and triplet excited states, observed in several triangle-shaped organic molecules containing conjugated carbon and nitrogen atoms, is an astonishing result that implies the breakdown of Hund’s rule. The phenomenon attracted interest for its potential toward triplet harvesting in organic LEDs. On a more fundamental vein, the singlet–triplet (ST) inversion sheds new light on the role of electron correlations in the excited-state landscape of π-conjugated molecules. Relying on the celebrated Pariser–Parr–Pople model, the simplest model for correlated electrons in π-conjugated systems, we demonstrate that the ST inversion does not require triangle-shaped molecules nor any specific molecular symmetry. Indeed, the ST inversion does not require strictly non-overlapping HOMO and LUMO orbitals but rather a small gap and a small exchange integral between the frontier orbitals.

## 1 Introduction

In closed-shell molecular systems, Hund’s multiplicity rule would locate the first excited singlet state S_1_ at higher energy than the corresponding triplet state T_1_, thus leading to a positive singlet–triplet (ST) gap Δ*E*_ST_. However, recent experimental^1^ and theoretical^2−10^ studies have shown that several triangle-shaped π-conjugated molecules (also called triangulenes) decorated with nitrogen atoms display a negative Δ*E*_ST_, thus violating Hund’s rule. Indeed, in these systems the highest occupied molecular orbital (HOMO) and the lowest unoccupied molecular orbital (LUMO) marginally overlap, and the lowest energy transition has a dominant multiresonant charge-transfer (MR-CT) character, with the electronic charge being transferred from the HOMO to the LUMO. In these conditions, the exchange integral, which is responsible for the ST splitting, is tiny, and the spin polarization correction, which is typically negligible, may be large enough to invert the ST gap.^11−15^

The family of MR-CT dyes is interesting in several respects. Even systems with the normal order of singlet and triplet states have small Δ*E*_ST_, making it possible to thermally populate the S_1_ state from the T_1_ state.^16−19^ Fluorescent MR-CT dyes have thus been intensively investigated as emitters featuring thermally activated delayed fluorescence (TADF). They are of special interest for organic light-emitting diode (OLED) applications, since the reduced polarity of the MR-CT state and the molecular rigidity guarantee very narrow and marginally solvatochromic emission spectra, properties in demand for emitters for (blue) OLEDs with high color purity.^1,2,20−31^ Here we will not address the family of MR-CT dyes at large but will focus on the specific issue of ST inversion. The theoretical interest in molecules breaking Hund’s rule is backed up by their applicative potential. The inverted ST order, in fact, allows for easy and efficient triplet harvesting in OLEDs, increasing their maximum internal efficiency from a disappointing 25% to a reassuring 100%.^20^

Spin polarization, the mechanism responsible for ST inversion, is due to electron correlation, so that conventional TD-DFT approaches to excited states are inadequate to address ST inversion, as was pointed out earlier.^2,32,33^ High-quality, computationally expensive methods have been exploited so far to deal with ST inversion, including CIS(D),^3,6^ SCS-CC2,^2,3,8,9^ EOM-CCSD,^2,4,5,34,35^ SCS-ADC(2),^2−4,7,8^ CASPT2,^2^ SA-CASSCF,^3,8^ SC-NEVPT2,^3,4,8^ and the Bethe–Salpeter equation in the GW approximation.^10^

In an effort to better understand the physics underlying the ST inversion, we undertook an extensive study of several molecular structures described by the celebrated Pariser–Parr–Pople (PPP) model Hamiltonian, the simplest model for correlated electrons in π-conjugated molecules. The model, proposed in the 1950s,^36−38^ successfully described the behavior of several families of small organic molecules.^37,39−47^ In the field of π-conjugated polymers, PPP was pivotal in understanding the anomalous behavior of polyacetylene,^48^ where correlated electrons are responsible for the appearance of a low-lying dark state, thus solving the puzzle of its nonfluorescent behavior. More recently, density matrix renormalization group (DMRG) approaches allowed to extend the PPP model to deal with graphene-based structures,^49^ to address singlet fission in polyenes,^50^ and to build models for dark states in polyene and carotenoid systems.^51^

Here we exploit the comparative simplicity of the PPP approach to understand ST inversion in triangulenes and related molecules. Comparing with exact diagonalization results, we critically discuss the validity of the double configuration interaction (CI) and related approaches, typically adopted to deal with *all-electron* models of triangulenes. The adopted PPP model cannot provide quantitatively accurate results for specific molecules. However, it is a solid and elegant approach that, applied here to a variety of molecular structures, discloses qualitative trends about the effects of electron correlation in π-conjugated molecules. The novel insights gained along these lines contribute to establish general and reliable guidelines toward the rational design of molecules featuring ST inversion. As an additional bonus, we show that information easily obtained at the Hartree–Fock (HF) level (including the HOMO–LUMO gap and exchange energy) offers safe guidance to recognize molecular structures with negative Δ*E*_ST_. An easy-to-use computational tool is made available for quick screening of interesting structures.

## 2 Theoretical and Computational Background

Much as in the Hückel model, the PPP model describes the physics of π electrons in planar conjugated molecules in terms of a minimal basis set that only accounts, on each atomic site, for just the p orbital orthogonal to the molecular plane. At variance with the Hückel model, PPP also accounts for the electron–electron (e–e) interaction in the zero differential overlap (ZDO) approximation. The PPP Hamiltonian reads:
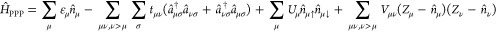
1where μ and ν run on the atomic sites,  is the Fermionic annihilation (creation) operator for an electron with spin σ in the atomic orbital (AO) on site μ, and  counts the total number of electrons on site μ. The first two terms of the above Hamiltonian are single–electron terms, where ε_μ_ measures the energy of the on-site orbital and *t*_μν_ is the hopping integral between sites μ and ν. As in the Hückel model, PPP only accounts for hopping integrals between sites connected by a σ bond. The remaining terms in the above Hamiltonian describe the e–e interaction: *U*_μ_ measures the repulsion between two electrons on the same site, *V*_μν_ is the repulsion between electrons on different sites, and *Z*_μ_ measures the charge on atom μ upon removal of the π electrons (*Z*_μ_ = 1 for carbon and aza nitrogen atoms; *Z*_μ_ = 2 for pyrrole nitrogens). We adopt the Ohno expression for *V*_μν_:^42,45,52^
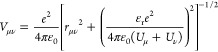
2where ε_r_ = 2 is the relative dielectric constant, as relevant to organic media.^45^

**Figure 1:**
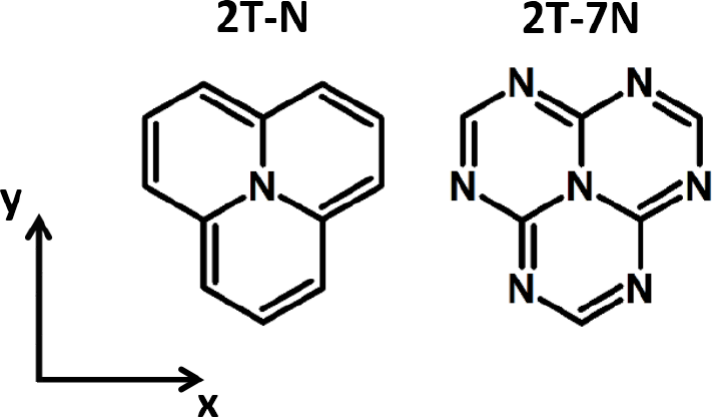
Sketches of the molecular structures of two prototypical triangulenes: 2T-N, also called cyclazine, and 2T-7N, also called heptazine.

The PPP Hamiltonian can be written on the so-called real space (RS) basis, comprising all states obtained by distributing the *n* π electrons on the *N* atoms. In the valence bond (VB) approach, the basis states are combined, following the Pauling rules, to be eigenstates of the total spin operator, , so that the Hamiltonian factorizes into subspaces having the same *S* value.^53,54^ The VB approach is extremely efficient, as it allows working with comparatively small matrices, but the VB basis is nonorthogonal and hence difficult to manage. On the other hand, in the RS representation, the basis states are selected as eigenstates of the *z*-component spin operator, . The RS subspaces are therefore much larger than the VB subspaces, but the basis set is orthogonal. For closed-shell systems, as discussed here, diagonalizing the PPP Hamiltonian in the *S*_*z*_ = 0 and 1 subspaces allows us to single out triplet (and higher-spin) states as those states that are present in both subspaces. The dimension of the RS basis grows fast with *N*, leading to very large Hamiltonian matrices that however are very sparse. Exploiting specific algorithms for the storage and diagonalization of sparse matrices,^55^ the PPP Hamiltonians for the 2T-N and 2T-7N molecules in Figure 1 can be diagonalized numerically, in spite of a basis that in the *S*_*z*_ = 0 subspace contains 2,944,656 states.

The RS diagonalization leads to exact *full-CI* results. However, in order to address larger systems and for a direct comparison with current quantum-chemical approaches to triangulene-like systems, we adopt a molecular orbital (MO) approach to the PPP Hamiltonian. Specifically, in the HF approximation the PPP Hamiltonian in eq 1 reduces to the one–electron Fock operator:
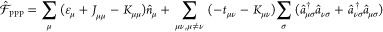
3where, in line with the PPP model, we exploited the ZDO approximation, so that the Coulomb operator has only diagonal terms,
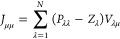
4while the exchange operator has elements
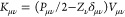
5In eqs 4 and 5, the matrix elements of the density operator are defined as
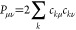
6where *k* runs on the occupied MOs in the ground-state configuration and *c*_*kμ*_ are the expansion coefficients of the *k*th MO on the AOs (see eqs S1 and S2). The HF Hamiltonian is solved self-consistently. Typically, 40–50 HF iterations are needed to reach convergence on the density matrix elements.

Once the HF MOs are obtained, as relevant to the ground-state configuration |g⟩, single, double, triple, etc. excited configurations are defined by transferring one, two, three, etc. electrons from the occupied to the virtual MOs. The PPP Hamiltonian is then written on the basis defined by the different configurations and diagonalized in the CI approach (see Supporting Information (SI) section S1). The PPP Hamiltonian on the CI basis is far less sparse than that on the RS basis, so that full CI is only feasible for very small molecules (up to ∼12 π electrons on 12 atoms). However, as will be shown below, the results converge quickly with the number of excitations, making it possible to obtain reliable results on fairly large systems.

The diagonalization of the CI Hamiltonian leads to the molecular excited states |f⟩, as needed to calculate the optical spectra. The electric dipole moment operator reads:
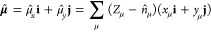
7where *x*_μ_ and *y*_μ_ are the Cartesian coordinates of site μ in the molecular plane. Absorption spectra are calculated assigning a Gaussian band shape to each transition:
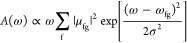
8where the sum runs over the excited eigenstates and the transition energies and dipole moments are ω_fg_ = *E*_f_ – *E*_g_ and , respectively (in the following, σ = 0.1 eV).

## 3 Results and Discussion

### 3.1 2T-N and 2T-7N as Reference Systems

**Figure 2:**
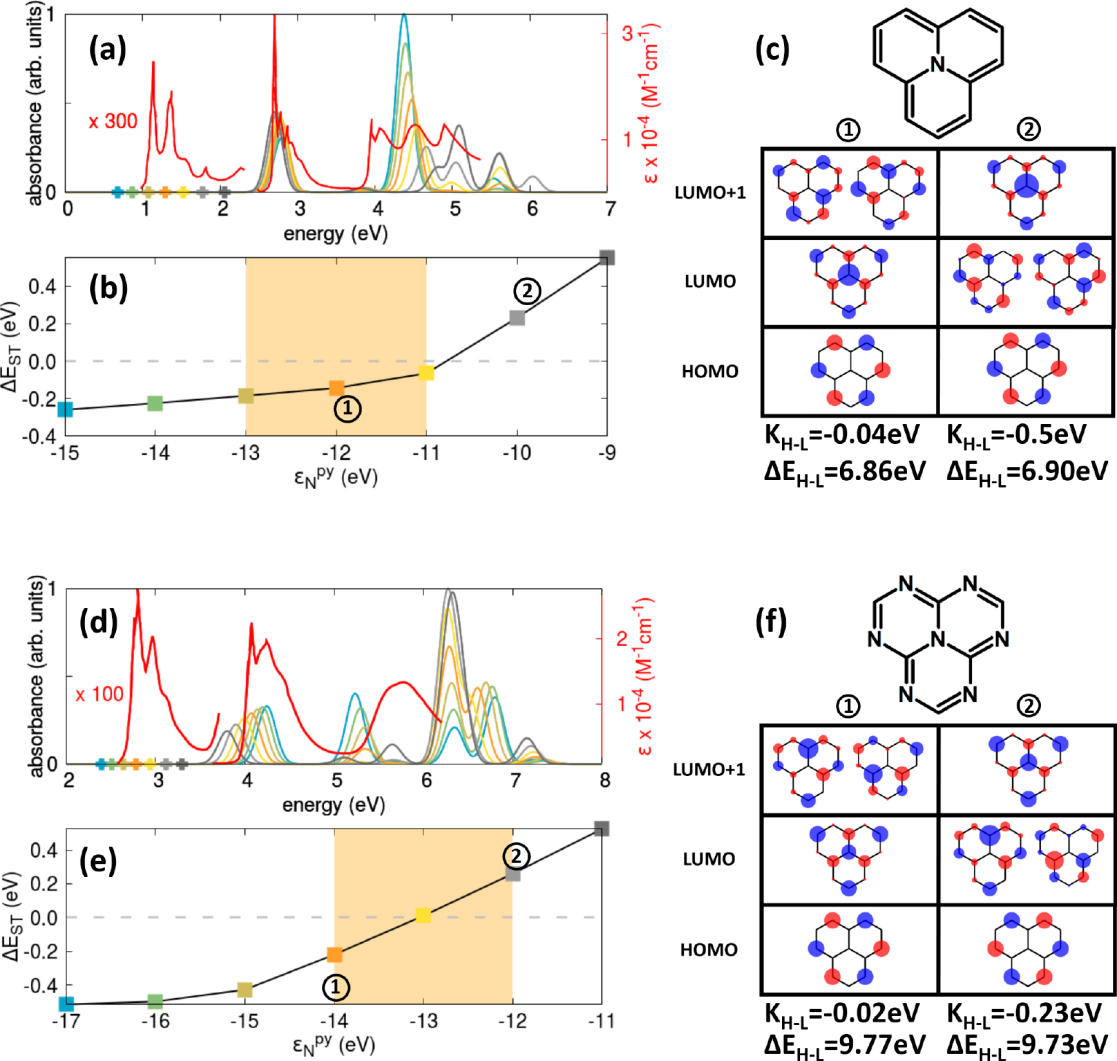
Simulating the photophysics of (a–c) 2T-N and (d–f) 2T-7N. (a) Absorption spectra of 2T-N. Red lines are experimental data from ref (57), and colored lines show results for several ε_N_^py^ values (same color code as in (b)). The crosses in the low-energy region mark the position of the dark state. The calculated spectra are normalized to the maximum absorbance of the most intense spectrum. (b) 2T-N singlet–triplet energy gaps calculated for different ε_N_^py^ values. The shaded area highlights ε_N_^py^ values giving acceptable agreement between calculated and experimental absorption spectra. (c) 2T-N frontier HF MOs for two values of ε_N_^py^, the corresponding exchange integral *K*_H–L_, and energy gaps Δ*E*_H–L_. (d) Absorption spectra of 2T-7N. Red lines are experimental data from ref (58), and colored lines show results for several ε_N_^py^ values (same color code as in panel (e)). The crosses in the low-energy region mark the position of the dark state. The calculated spectra are normalized to the maximum absorbance of the most intense spectrum. (e) Same as (b) for 2T-7N. (f) Same as (c) for 2T-7N.

**Figure 3:**
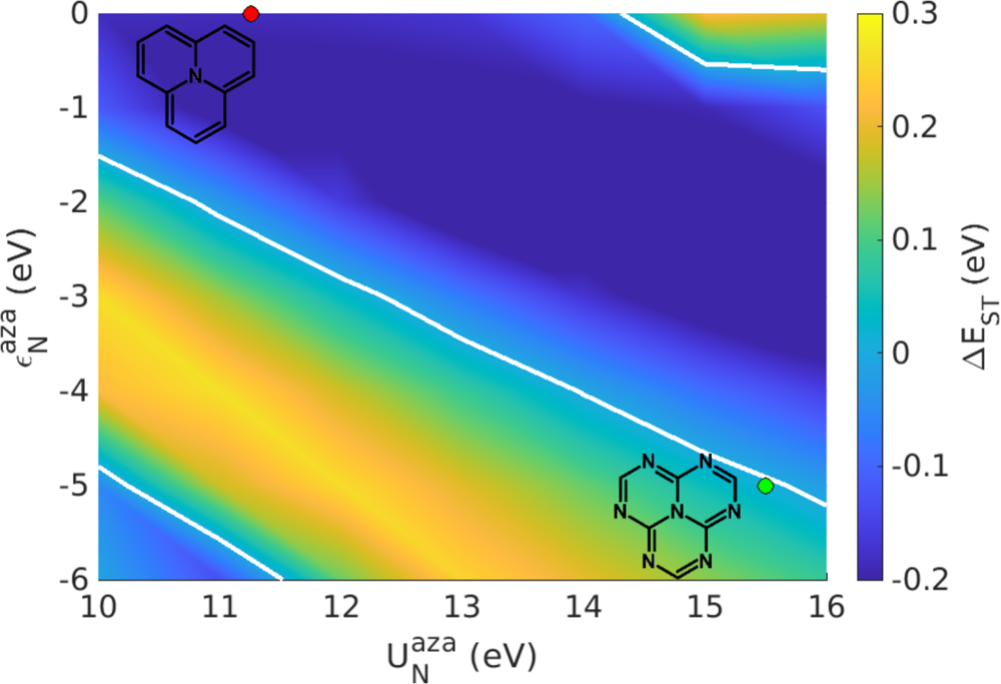
Color map showing the dependence of the ST gap (color code on the right) on ε_N_^aza^ and *U*_N_^aza^, with ε_N_^py^ = −13 eV. White lines at Δ*E*_ST_ = 0 mark the boundary between the ST-inverted region (dark blue) and the normal region (light blue and yellow). Red and green dots indicate the parameters relevant to 2T-N and 2T-7N, respectively. The calculations were done at the PPP CISDT level with model parameters defined in the main text.

To start, we target the two triangulene structures in Figure 1. In recent years, these molecules have been extensively investigated theoretically as prototypical examples of ST inversion.^2,7,32,56^ Experimental absorption spectra of both dyes, collected in the 1980s,^57,58^ will be exploited to parametrize and validate the PPP model. The PPP parameters for C atoms, derived in the 1950s,^37^ are well-established and transferable among different molecules.^42,45,53,59^ Specifically, setting the on-site energy of C atoms to zero, we adopt the standard values for the on-site e–e interaction, *U*_C_ = 11.26 eV, and for nearest-neighbor C–C hopping, *t* = 2.4 eV. For nitrogen atoms, a unique set of PPP parameters is not available.^41−45,53,60−66^ We set the hopping integral for C–N bonds to the same value as for C–C bonds.^53^ Typically, the on-site e–e repulsion is slightly larger for N atoms than for C atoms.^45^ Best agreement with experimental data is obtained here by setting *U*_N_ ∼ 15 and 15.5 eV for pyrrole and aza nitrogens, respectively. These choices marginally affect the results (see SI section S2). For the molecular geometry, for the sake of simplicity, we set all angles to 120° and all bond lengths to 1.4 Å (results for different geometries are marginally different; see Figure 2S). The most delicate issue is the on-site energy of the N atoms. We fix the site energy of the aza nitrogen at ε_N_^aza^ = −5 eV and adjust the pyrrole nitrogen site energy ε_N_^py^ to best simulate the experimental spectra (Figure 3S shows results obtained for different ε_N_^aza^ values).

Experimental absorption spectra of 2T-N and 2T-7N from refs (57) and (58) are shown as red lines in Figure 2a,d, respectively. For both molecules, the lowest-energy absorption band is very weak and is ascribed to a forbidden transition that gains some intensity through vibronic coupling. This dark transition is located at ∼1–1.5 and ∼2.6–3 eV for 2T-N and 2T-7N, respectively. An intense and sharp transition is seen in 2T-N at ∼2.7 eV, followed by a broad absorption for energies higher than ∼3.9 eV. The 2T-7N spectrum is similar but blue-shifted, with the first intense absorption observed at 4–4.3 eV and a second broad absorption at energies higher than ∼5.3 eV. In the same figure, superimposed on experimental spectra, we show spectra calculated accounting for up to triple excitations (i.e., CISDT), to ensure converged results (see below). Results are shown for different values of the on-site energy of the triangulene central nitrogen, ε_N_^py^. For both molecules, the lowest-energy state, S_1_, is a dark state (A_2_^′^ symmetry in the *D*_3*h*_ point group), and its location is marked by a cross in the figure.

For 2T-N (Figure 2a), upon increasing the electron-withdrawing nature of the central N atom (i.e., for more negative ε_N_^py^), the dark S_1_ state moves to the red. Similarly, the highest-energy excitation red-shifts, while the band at intermediate energy, ascribed to S_2_, a doubly degenerate E′ state, is marginally affected by ε_N_^py^. For 2T-N, acceptable agreement with experiment is observed in the region −13 eV < ε_N_^py^ < −11 eV. Quite interestingly, Figure 2b shows that in this region (highlighted in orange) the ST gap is consistently negative.

Moving to 2T-7N (Figure 2d), the dark state again moves to the red when ε_N_^py^ becomes more negative, but at variance with 2T-N, the intermediate S_2_ ← S_0_ transition is also affected by ε_N_^py^, with the corresponding peak moving to the blue when ε_N_^py^ becomes more negative. An acceptable agreement with experiment is obtained for ε_N_^py^ values ranging from −14 to −12 eV (the region highlighted in orange in Figure 2e). So, we will stick to ε_N_^py^ = −13 eV as a good compromise to describe both 2T-N and 2T-7N. In the following, when not otherwise stated, results will be shown for this value of ε_N_^py^. Quite interestingly, the sign of the ST gap of 2T-7N in the relevant region goes from negative values, Δ*E*_ST_ = −0.22 eV at ε_N_^py^ = −14 eV, to positive values, Δ*E*_ST_ = +0.26 eV at −12 eV. Accordingly, the ST inversion, safely assessed in 2T-N, is marginal in 2T-7N.

The HF MOs of 2T-N and 2T-7N in Figure 2c,f, respectively, give interesting clues. When the ST gap is negative (points ① in Figure 2b,e), the HOMO and LUMO are localized on two complementary sublattices, and the HF HOMO–LUMO exchange energy *K*_H–L_ is as low as −0.04 eV (2T-N) and −0.02 eV (2T-7N). In either case, the LUMO+1 orbital is doubly degenerate and is clearly not complementary to the HOMO. When Δ*E*_ST_ becomes positive (points ② in Figure 2b,e), the nature of the LUMO and LUMO+1 is reversed, so that the HOMO–LUMO complementarity is lost, and sizable *K*_H–L_ values are calculated (−0.5 eV for 2T-N and −0.23 eV for 2T-7N). We stress that this notable reversal in the natures of the LUMO and LUMO+1 is not shown by the Hückel MOs, demonstrating once again that electron correlations are crucial for ST inversion.

The different behavior observed for 2T-N and 2T-7N in terms of Δ*E*_ST_ reflects the diverse natures of the triangulene rim in the two cases. In Figure 3, we show the dependence of the ST gap on the parameters that define the triangulene rim, namely, ε_N_^aza^ and *U*_N_^aza^. The red dot on the map corresponds to the 2T-N parameters, where the rim is only composed of C atoms. If, starting from this position, we decorate the odd sites on the rim with electron-withdrawing atoms, with progressively more negative on-site energy, the ST inversion is lost when ε_N_^aza^ becomes more negative than about −2.1 eV. However, upon increasing the on-site electron repulsion from the value relevant to C atoms to that relevant to N atoms, the ST inversion is favored. 2T-7N, with its six aza nitrogens on the rim, is precisely on the verge of the ST-inverted region (green dot on the map). This last observation contrasts with the results of many all-electron calculations that typically predict a more pronounced ST inversion in 2T-7N than in 2T-N.^2−4,6,30,67,68^ Of course, the PPP model is not an exact model, and the results are affected by the specific choice of the molecular geometry and model parameters (see SI section S2 for specific illustrative cases). However, it is important to stress that widely adopted all-electron calculations are by themselves approximate. Just as an example, large uncertainties in π → π* transition energies are estimated (e.g., ∼0.11 eV for CCSD and ∼0.17 eV for ADC(2)),^69,70^ making the error in Δ*E*_ST_ of the same order of magnitude as the calculated gap itself. On the other hand, the computational cost of the more precise CC3 approach makes the calculation prohibitive for such large systems.

**Figure 4:**
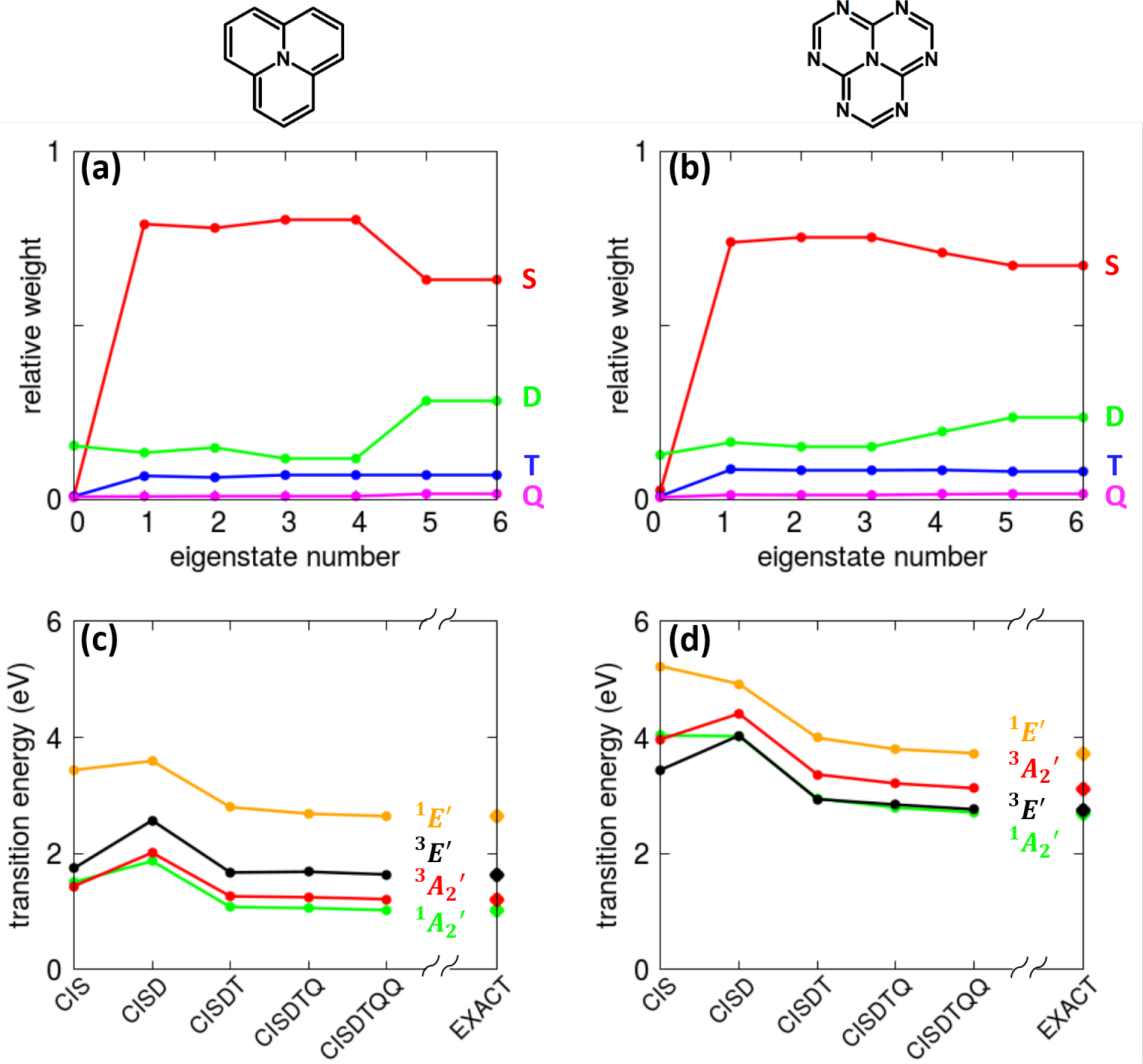
Relative weights of single (S), double (D), triple (T), and quadruple (Q) excitations for the first seven singlet and triplet eigenstates of (a) 2T-N and (b) 2T-7N calculated at the CISDTQ level, together with the excitation energies of the first few electronic states of (c) 2T-N and (d) 2T-7N at different levels of theory. Relevant results from exact diagonalization in the real space basis (full CI) are shown as colored dots in (c) and (d). Model parameters are defined in the main text.

Indeed, we are in the position to address the reliability of commonly adopted all-electron quantum-chemical calculations while at the same time validating our approach. As for 2T-N and 2T-7N, all-electron calculations are typically truncated to single and double electronic CIs. Among them, we mention CIS(D), an approach that accounts for single CI, and introduces double CI perturbatively; spin-component-scaled second-order coupled cluster (SCS-CC2), spin-component-scaled second-order algebraic diagrammatic construction (SCS-ADC(2)),^2,3,7,56^ double-hybrid DFT,^68^ and DFT multireference configuration interaction (DFT-MRCI).^71^ All these approaches confirm the important role played by double CI to properly describe the ST inversion. However, the fast increase in the number of multiple CI precludes higher-order studies using all-electron wavefunction- or density-based approaches. The PPP model, with its minimal representation of π electrons, lends itself quite naturally to explore higher-order CIs. In Figure 4 a,b, we show the total weights of single (S), double (D), triple (T), and quadruple (Q) CIs in the lowest seven eigenstates of 2T-N and 2T-7N. For both molecules, as expected, single and triple CI marginally contribute to the ground state (according to the Brillouin theorem, single and triple CI are not directly mixed to the ground-state configuration and marginally contribute to it via their indirect mixing to double CI). For excited states, in both molecules double and triple CIs enter the picture with similar and sizable weights. This is a strong indication that a reliable approach to the excited states of these molecules should include at least triple CIs. On the other hand, the weight of quadruple CIs stays very small, suggesting that fourth- and higher-order CIs can be safely neglected. Similarly, the Hartree–Fock ground state with its doubly occupied HOMO plays no role in the first few excited states (see SI section S3).

Truncating the expansion at the CISD level, the ground state is stabilized, but the stabilization of the excited states due to the triple CI contribution is missing. As a result, CISD transition energies are overestimated, a spurious effect that is fixed when triple excitations are accounted for (see panels (c) and (d)). This result is fully confirmed by the comparison with RS exact diagonalization transition energies, which perfectly match with the CISDT results. However, reaching triple CI in all-electron quantum-chemical calculations is extremely demanding. Most often, CIS(D) approaches, where double CIs are introduced perturbatively, seem to provide reasonable results as for transition energies. However, caution is needed since the wavefunctions stay at the CIS level. On the other hand, CIS(D) introduces perturbative corrections ranging from ∼1 to ∼2 eV on the singlet states of 2T-N and 2T-7N (see SI section S4 for ab initio CIS(D) results), shedding doubts on the reliability of a perturbative treatment of these two molecules.

Figure 4 tells us another interesting story. In either 2T-N and 2T-7N, the singlet and triplet excited states with MR character, ^1^A_2_^′^ and ^3^A_2_^′^, respectively, have the normal order (positive ST gap) at the CIS level, but as soon as correlations enter into play, from CISD up, their order is inverted, with the triplet MR state (^3^A_2_^′^) lying at an energy higher than the relevant singlet ^1^A_2_^′^. However, there is another pair of states, ^3^E′ and ^1^E′, whose energies are not too far. In particular, in the case of 2T-7N, the two triplets ^3^A_2_^′^ and ^3^E′ invert their energies. In this situation, while the relative energy of the two MR states would still give a negative ST gap, the presence of another non-MR triplet at low energy makes the ST inversion critical.

### 3.2 ST Inversion: An Exploratory Study

**Figure 5:**
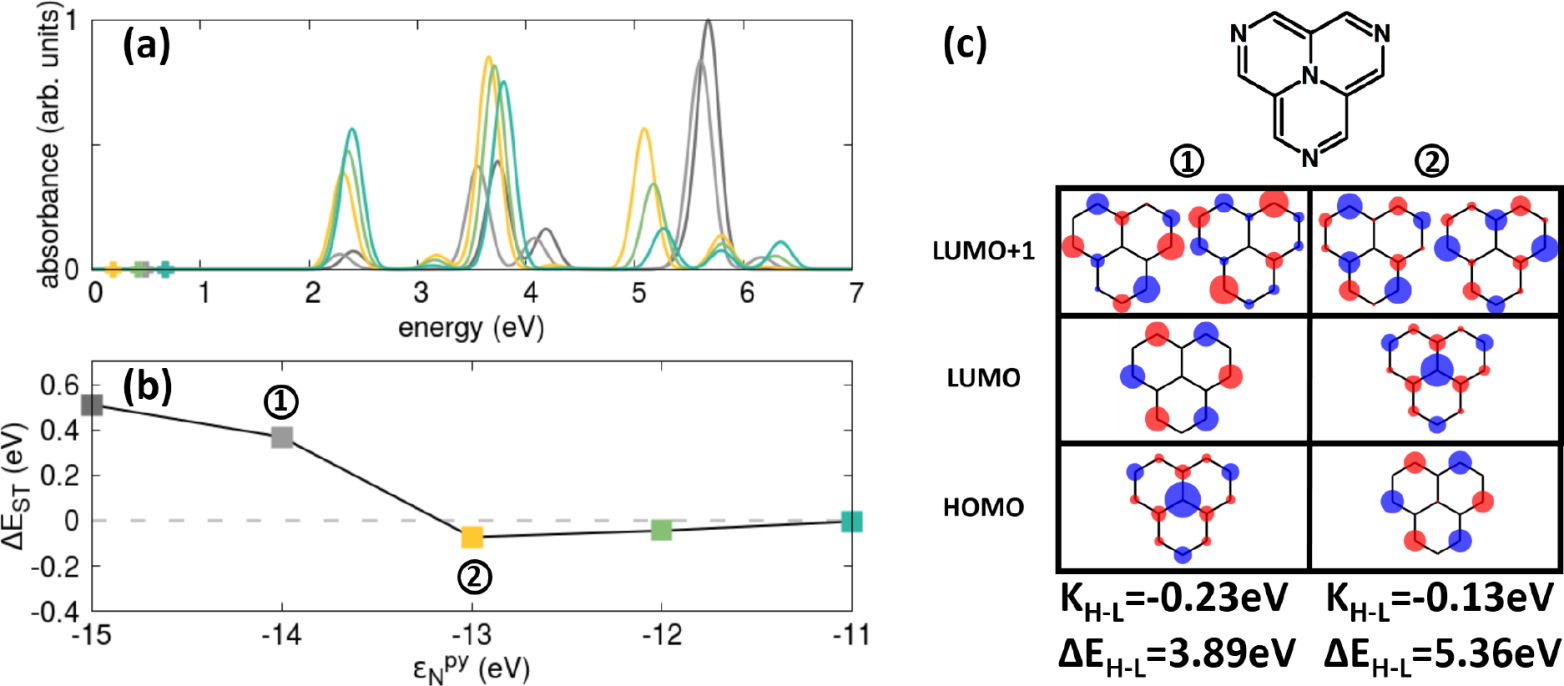
Effect of ε_N_^py^ on the energy levels of 2T-4N. (a) Absorption spectra calculated for different ε_N_^py^ values (the same color code is used in (b)). The crosses mark the position of the first excited (dark) state. Spectra are normalized with respect to the maximum absorbance of the most intense spectrum. (b) Calculated singlet–triplet energy gaps. (c) Frontier Hartree–Fock MOs obtained for two different ε_N_^py^ values. Relevant HF HOMO–LUMO exchange energies *K*_H–L_ and energy gaps Δ*E*_H–L_ are also reported. Other parameters are defined in the main text. Results were obtained at the PPP CISDT level.

PPP is a reliable and computationally inexpensive approach to π-conjugated molecules that, easily allowing the exploration of a large number of structures, helps to shed light on the physics of the ST inversion. We start our analysis with 2T-4N (Figure 5), a molecule having the same *D*_3*h*_ symmetry as 2T-N and 2T-7N but qualitatively different behavior for the ST gap. As in 2T-N and 2T-7N, the lowest-energy transition is dark. The allowed transitions move first to the red when the orbital on the central N atom is stabilized (ε_N_^py^ going from −11 eV to −13 eV), and then blue-shift for further stabilization of the central N orbital (−14 eV > ε_N_^py^ > −15 eV), as shown in Figure 5a. More interestingly, in this case, at variance with either 2T-N or 2T-7N, if the central nitrogen orbital is stabilized (i.e., ε_N_^py^ is made more negative), the ST gap becomes positive (see panel (b)) and the HOMO/LUMO nature is switched (see panel (c)). Indeed, for 2T-4N, the HOMO and LUMO show a tiny overlap, thus leading to a fairly large exchange energy, *K*_H–L_ ∼ −0.1 eV, reducing the MR character of the low-lying excitation. As a result, the ST inversion region is strongly suppressed, and the calculated ST gap is only marginally negative.

The molecules discussed so far, 2T-N, 2T-7N, and 2T-4N, all belong to the *D*_3*h*_ point group, and the question arises whether the ST inversion is somehow related to the molecular symmetry. This is an important point, since in these three molecules the lowest excited singlet is a dark (i.e., optically forbidden) state. These molecules are therefore very weakly fluorescent, so that, in spite of the ST inversion, they are of little interest for OLED applications. Upon lowering the symmetry, the S_1_ state can acquire oscillator strength, and if the ST inversion is maintained, extremely promising fluorescent dyes for an OLED with 100% internal quantum efficiency could be devised.

**Figure 6:**
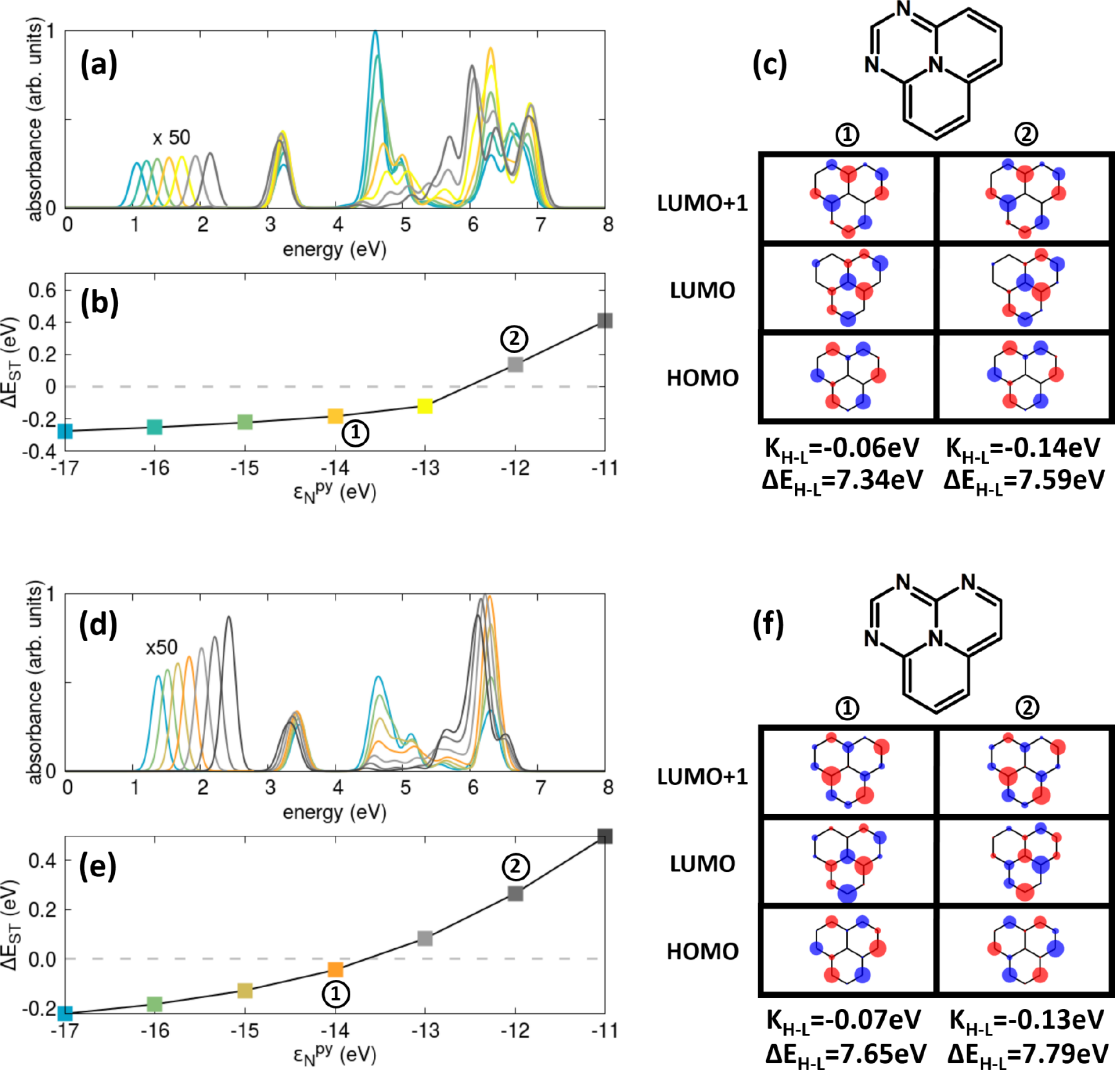
Decorating the 2T-N rim with an increasing number of aza nitrogen atoms while changing the pyrrole nitrogen site energy. Calculated (a, d) absorption spectra, (b, e) ST gaps, and (c, f) HF frontier orbitals are shown for 2T-3N and 2T-4N′. In (a) and (d), spectra are normalized with respect to the maximum absorbance of the most intense spectrum, while the band relevant to the first excited singlet state is magnified by 50 times. In (c) and (f), we report the relevant HOMO–LUMO exchange energies, *K*_H–L_, and gaps, Δ*E*_H–L_. Calculations were done at the PPP CISDT level for the same parameters used in Figure 4.

To address this issue, in Figure 6 we decorated the external rim of 2T-N with a variable number of N atoms. The two molecules, 2T-3N and 2T-4N′ (structural isomer of the system in Figure 5) have *C*_2*v*_ and *C*_*s*_ symmetry, respectively, so that degenerate states are not possible. Comparing with Figure 2a, as relevant to 2T-N, all spectral features move to the blue upon increasing the number of N atoms in the external rim. Most importantly, the lowest-energy transition, strictly forbidden in either 2T-N or 2T-7N, acquires sizable intensity. Quite interestingly, despite the lack of complementarity between the HOMO and LUMO, the exchange energy is fairly small when the electron-withdrawing nature of the central N atom is large. As a result, a region with ST inversion is obtained for both molecules at a large negative ε_N_^py^. Indeed, the best descriptor for ST inversion is not the complementarity of the frontier orbitals but rather the value of the HOMO–LUMO exchange energy.

In a recent work,^72^ the simultaneous presence of a *C*_2_ axis and σ_v_ plane was suggested as essential to ST inversion, thus making *C*_2*v*_ the lowest-symmetry point group of molecules breaking the Hund’s multiplicity rule. Indeed, results on 2T-4N′ in Figure 6 show that comparatively large and negative Δ*E*_ST_ values can be achieved even in a system where the only symmetry element is the molecular plane but where the noncomplementary frontier orbitals do minimize the HOMO–LUMO exchange integral.

**Figure 7:**
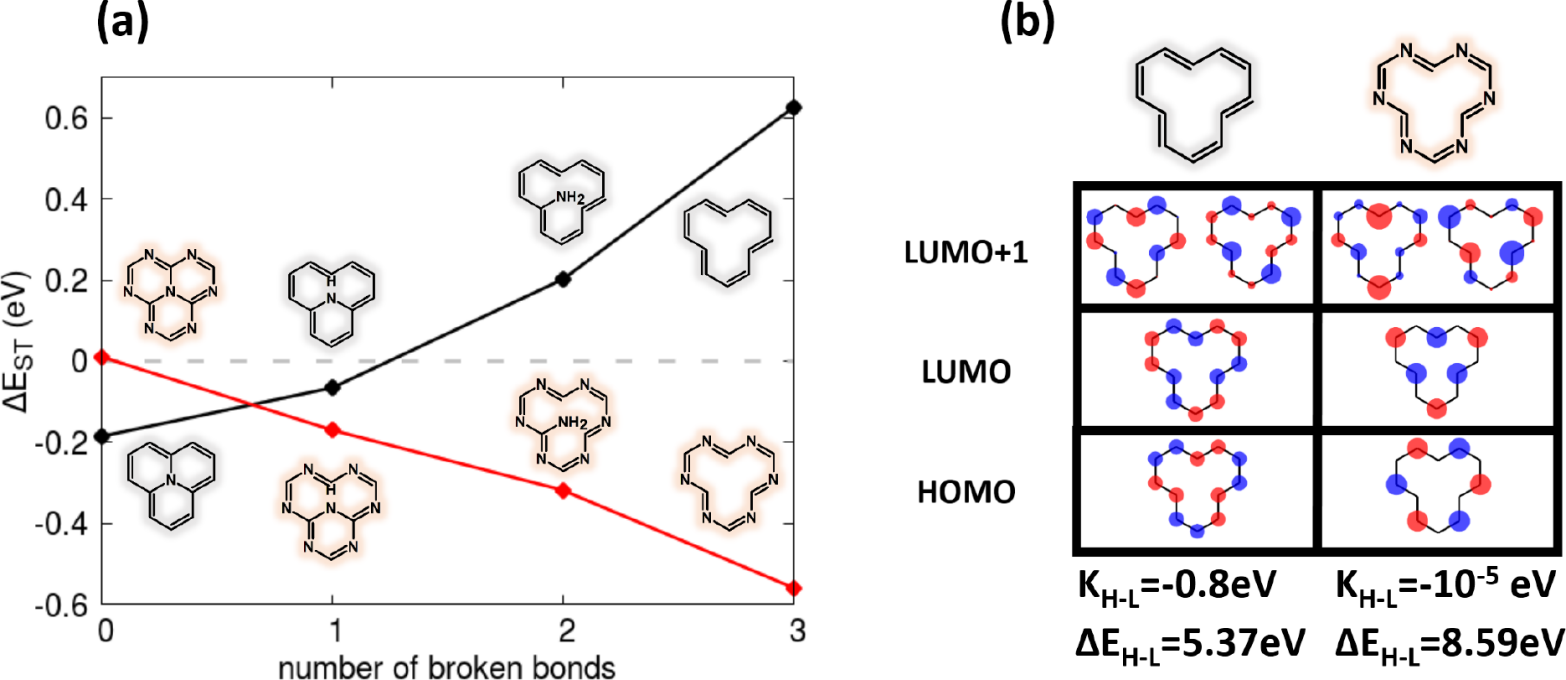
Role of central connectivity in the ST gap. (a) Calculated ST gaps as functions of the number of broken central bonds for 2T-N (black curve) and 2T-7N (red curve). Calculations were done at the PPP CISDT level. (b) Frontier HF MOs for the two annulenes obtained by breaking all three central bonds in 2T-N and 2T-7N. Relevant HOMO–LUMO exchange energies and gaps are also shown. The same model parameters as in Figure 4 are used.

Breaking some of the bonds that connect the central N atom in 2T-N and 2T-7N to the rim, as shown in Figure 7, is another way to reduce the molecular symmetry. Quite interestingly, breaking bonds in 2T-N quickly drives the molecule from the region with inverted ST order to the normal region, while the opposite occurs for 2T-7N. As expected, the lowest-lying singlet state acquires sizable intensity when one or two bonds are broken and the symmetry of the system is reduced. Of course, when all bonds are broken, the *D*_3*h*_ symmetry is recovered, and the lowest-energy states are dark (see SI section S5).

Possibly the most surprising result from Figure 7 is the observation that just the 2T-7N rim, without the central N atom, has the largest negative ST gap, suggesting that the triangulene structure itself is not really needed to observe the ST inversion. On the other hand, coming back to our prototypical dyes 2T-N and 2T-7N (Figure 2), it is clear that the ST inversion is favored when the on-site energy on the central N is more negative, i.e., in systems where the two electrons from the central N are marginally involved in the rim physics. Figure 7b shows the HF frontier orbitals calculated for the cyclododecahexaene and hexaazacyclododecahexaene molecules, corresponding to the rims of the 2T-N and 2T-7N molecules, respectively. In cyclododecahexaene, the lack of spatial separation between the HOMO and LUMO is reflected in a large exchange energy: the molecule (a simple cyclic polyene) does not qualify as an MR-CT dye. On the other hand, the frontier orbitals of the hexaazacyclododecahexaene variant, with their small *K*_H–L_ value, confirm its MR-CT nature.

### 3.3 ST Inversion: A Fresh View of Spin Polarization

**Figure 8:**
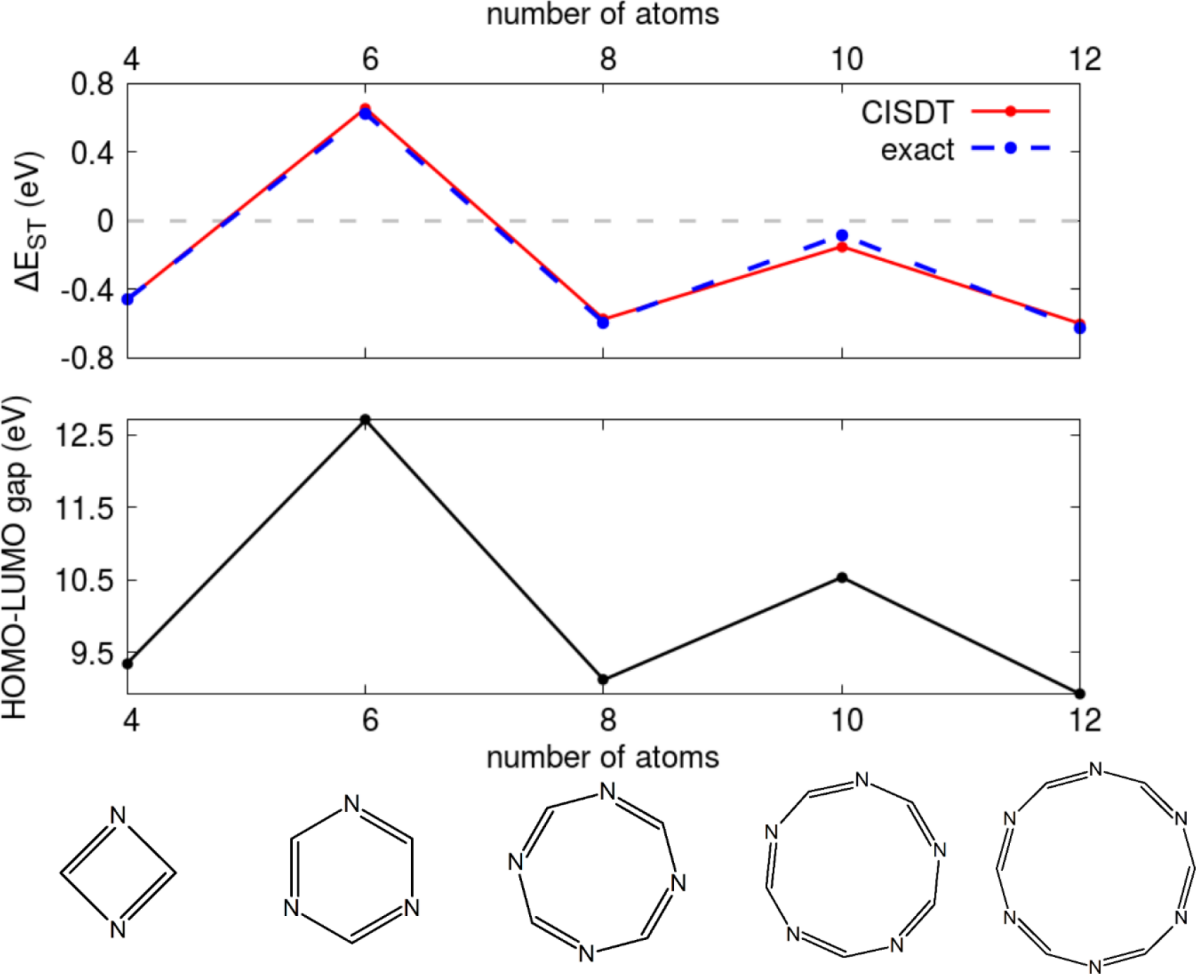
Results for rings with different numbers of aza nitrogen atoms. Upper panel: ST gap values. The red curve refers to the PPP CISDT results. The dashed blue line refers to results from exact diagonalization on the RS basis (full CI). Lower panel: HF HOMO–LUMO gap for the different aza-doped rings. The same parameters as in Figure 4 are used.

Figures 6 and 7 suggest that the observation of an inverted ST gap is not intrinsically related to the symmetry of the molecule or to the presence of the connectivity of *triangulene-like* structures, but rather, it is amplified in (CHN)_*x*_ rings. (CHN)_*x*_ rings are a very useful playground to understand the phenomenon of dynamical spin polarization, which is pivotal to ST inversion. Dynamical spin polarization was discussed earlier in the framework of π-electron models,^11−15,73^ demonstrating very clearly that electron correlation governs ST inversion but that at least double excitations are needed to capture the phenomenon. To shed light on the elusive dynamical spin polarization issue, we performed calculations on (CHN)_*x*_ rings by putting the atoms at the vertices of regular polygons with bond lengths equal to 1.4 Å. Figure 8 shows the evolution of the singlet triplet gap and the HF HOMO–LUMO gap for rings with a variable number of atoms from 4 to 12. A couple of observations are in order. As expected, triple-CI results are basically equivalent to full-CI results, at variance with double-CI results (see Figure 7S). The adopted idealized geometry is not realistic for all structures, but for the triazine molecule (i.e., *x* = 3), whose equilibrium geometry corresponds to an (almost) regular hexagon.^69,70^ For this specific molecule, it is therefore interesting to compare the PPP ST gap value, ∼0.65 eV, with available results from all-electron CC3 calculations, pointing to a positive ST gap, ∼0.9 eV. The agreement is far from perfect but definitely acceptable for a semiempirical approach. On the other hand, results reported in SI section S7 for (CHN)_2_ and (CHN)_3_ confirm that CC2 results are not accurate enough. Overall, PPP results, while not quantitatively correct, compare favorably with CC3 results, which show a negative gap for (CHN)_2_ in either the idealized or optimized geometry and a positive gap for (CHN)_3_ (see SI section S7).

While the adopted idealized geometries are not realistic for most rings, the results in Figure 8 are extremely interesting, as they demonstrate an almost perfect correlation between the HF HOMO–LUMO gap and the ST gap. The 4*n*/4*n* + 2 alternancy of the HOMO–LUMO gap is a clear remnant of the well-known alternancy of cyclic polyenes. Indeed, in cyclic polyenes, either at the Hückel or HF level, the HOMO–LUMO gap is sizable for 4*n* + 2 rings but vanishes for 4*n* rings. In (CHN)_*x*_ rings, a splitting of the HOMO–LUMO gap is found also in 4*n* rings, but it stays lower than for 4*n* + 2 rings (see Figure 6S). In all cases, the HOMO–LUMO gap decreases with an increase in the ring dimension.

We are now in the position to understand the correlation between the ST and HOMO–LUMO gaps in Figure 8. The lowest excited singlet and triplet states in our closed-shell systems are dominated by configurations where one electron is transferred from the HOMO to the LUMO. The exchange energy, which in most systems is responsible for triplet states lying lower than singlets, is negligible in these alternating rings (see SI section S8), and the typically tiny effect of dynamical spin polarization may enter into play, inverting the ST gap. Specifically, the singlet excited state is stabilized by the interaction with the doubly excited state, where both electrons reside on the LUMO. This stabilization is not possible for the corresponding triplet states. In close analogy with the kinetic exchange energy stabilization in Heisenberg antiferromagnets,^74^ a perturbative estimate of the singlet stabilization due to spin polarization is given by . Figure 8, showing an impressive correlation between the size of the HOMO–LUMO gap and the ST gap, neatly confirms this view: the singlet stabilization is larger the smaller the HOMO–LUMO gap is, and the only ring with a positive ST gap is triazine (CHN)_3_, with the largest HOMO–LUMO gap. The picture is further confirmed by our results for 2T-N and 2T-7N, whose ST gaps, about −0.2 and 0 eV, respectively, can be related to a much smaller HOMO–LUMO gap (∼6.9 eV) for 2T-N than for 2T-7N (∼9.8 eV). In the end, despite the adopted idealized geometries, the rings in Figure 8 show how a molecular edge with alternating electron-withdrawing and electron-donor groups is a key ingredient for ST inversion.

## 4 Conclusions

The inversion of the lowest excited singlet and triplet states involves breaking Hund’s rule and deserves some explanation. Typically, triplets have lower energy than the corresponding singlets because of the exchange energy. Accordingly, the first requirement for ST inversion is a small exchange energy between the HOMO and LUMO. To this end, a possible strategy consists in having non-overlapping HOMO and LUMO, so that the two orbitals have sizable weight on two different subsets of atoms. Systems with disconnected HOMO and LUMO belong to a large and interesting family of MR-CT dyes. However, our results show quite explicitly that a perfectly disjointed nature of HOMO and LUMO is not a strict prerequisite for ST inversion. A more reliable indication of the propensity to break Hund’s rule is recognized in the HOMO–LUMO exchange energy itself.

To observe ST inversion, spin polarization is needed to bring the energy of the lowest excited singlet below the energy of the lowest triplet in systems with a small HOMO–LUMO exchange energy. Spin polarization is driven by electron correlations and cannot be modeled in single-CI approaches. Accordingly, TD-DFT, arguably the most widely applied quantum-chemical approach to excited states in molecular systems, fails to describe ST inversion, which requires at least double excitations. By exploiting the PPP model, we investigated the role of higher-order CIs, showing that reliable results on inverted ST systems can only be obtained if at least triple excitations are accounted for (see Figure 4).

We studied several triangulene-based molecules to grasp the physics of ST inversion. Starting from the highly symmetric and very weakly fluorescent 2T-N, 2T-7N (see Figure 2), and 2T-4N (see Figure 5), we moved to fluorescent 2T-3N and 2T-4N′ (see Figure 6), obtained by decorating the edge of 2T-N with two and three N atoms, respectively. In all cases, we found non-negligible regions of the parameter space showing ST inversion. We demonstrated that neither the presence of the *C*_2_ axis and σ_v_ plane nor the very specific connectivity of triangulenes is actually needed for ST inversion.

The key ingredient for a negative singlet–triplet gap is a molecular edge with alternating electron-donor (D) and electron-acceptor (A) groups. In 2T-N, where the rim is made of 12 carbon atoms, the central nitrogen creates an alternating pattern of electron-poor and electron-rich sites on the rim. Accordingly, upon gradually breaking the bonds that connect the central nitrogen to the edge until the central nitrogen is completely decoupled from the rim, one recovers a cyclic polyene structure and then loses the ST inversion (cf. Figure 7). In 2T-7N, instead, the aza nitrogens along the rim create by themselves an alternating pattern and the central nitrogen atom is not actually needed to observe ST inversion. Rather, in 2T-7N the central N atom is detrimental to ST inversion, putting 2T-7N in the difficult region where determining the sign of the ST gap is tricky, as it fluctuates depending on the specific choice of model parameters (Figure 3). In the PPP π-electron-only picture, large (CHN)_*x*_ rings in an idealized planar and regular geometry are the most promising structures for ST inversion.

Triple-CI models, which are needed to properly deal with ST inversion, are computationally very demanding, making their exploitation in explorative studies prohibitive. However, our analysis demonstrates that good indicators for ST inversion can already be obtained at the HF level. Specifically, a computationally inexpensive (ground state) calculation of the molecular MOs gives access to both the HOMO–LUMO gap and the relevant exchange integral. Systems with small exchange integrals and small gaps are promising for ST inversions and should be considered for additional theoretical analysis or for experimental work. An easy-to-use (PPP-based) computational tool is made publicly available (see SI section S8) to offer the synthetic chemist a very easy and computationally inexpensive approach to identify the most promising molecular structures with an inverted ST gap (https://github.com/francescodimaiolo/Hartree-Fock_PPP_tool).

## Data Availability

The data that support the findings of this study are available from the corresponding author upon reasonable request. An easy-to-use (PPP-based) computational tool is made publicly available for the calculation of *K*_H–L_ (https://github.com/francescodimaiolo/Hartree-Fock_PPP_tool).

## Supporting Information Available

Derivation of the PPP model on the MO basis (section S1); additional results obtained for different model parameters values and different molecular geometries (i.e., angles and bond lengths) (section S2); relative weights of Hartree–Fock ground state and first few excitations in the lowest seven eigenstates of 2T-N and 2T-7N (section S3); ab initio CIS(D) results for 2T-N and 2T-7N (section S4); optical spectra calculated for the systems in Figure 7 (section S5); additional results for (CHN)_*x*_ rings (section S6); validation of PPP results against ab initio calculations for (CHN)_2_ and (CHN)_3_ (section S7); HOMO–LUMO exchange integral expression on the MO and AO basis (section S8) (PDF)
