# Supplementary material for: Shining Light on Inverted Singlet–Triplet Emitters
Source: J Chem Theory Comput. 2023 Nov 22;20(2):902–13. doi: 10.1021/acs.jctc.3c01112 (PMC10809715; doi:10.1021/acs.jctc.3c01112)
Supplement: Supplementary file 1 — ct3c01112_si_001.pdf [file ct3c01112_si_001.pdf]

Supplementary Information

Shining light on inverted singlet-triplet emitters

Matteo Bedogni, Davide Giavazzi, Francesco Di Maiolo,* and Anna Painelli

Department of Chemistry, Life Science and Environmental Sustainability, Università di Parma, 43124 Parma, Italy

E-mail: francesco.dimaiolo@unipr.it

S1 The PPP Hamiltonian on the MO basis

The operators $\hat{b}^{(\dagger)}_{k\sigma}$ that annihilate (create) an electron with spin σ in the k-th molecular orbital (MO) can be written as linear combination of the $\hat{a}^{(\dagger)}_{\mu\sigma}$ (on-site) operators:

$$\hat{b}_{k\sigma} = \sum_{\mu} c_{\mu,k}\hat{a}_{\mu\sigma} \tag{1}$$

$$\hat{b}^{\dagger}_{k\sigma} = \sum_{\mu} c_{\mu,k}\hat{a}^{\dagger}_{\mu\sigma} \tag{2}$$

where the expansion coefficients are obtained upon diagonalization of the Fock operator in Eq. 3, main text. With this transformation, the PPP model Hamiltonian in Eq. 1, main text is rewritten on the MO basis as:[1]

$$\begin{aligned}
\hat{H}^{CI}_{PPP} &= \sum_{ij}\sum_{\sigma}\left(\sum_{\mu}\varepsilon_{\mu}c_{\mu,i}c_{\mu,j}\right)\hat{b}^{\dagger}_{i\sigma}\hat{b}_{j\sigma} \\
&- t\sum_{ij}\sum_{\sigma}\left[\sum_{\mu\nu,\mu\neq\nu}(c_{\mu,i}c_{\nu,j}+c_{\nu,i}c_{\mu,j})\right]\hat{b}^{\dagger}_{i\sigma}\hat{b}_{j\sigma} \\
&+ \sum_{ijkl}\left(\sum_{\mu}U_{\mu}c_{\mu,i}c_{\mu,j}c_{\mu,k}c_{\mu,l}\right)\hat{b}^{\dagger}_{i\uparrow}\hat{b}_{j\uparrow}\hat{b}^{\dagger}_{k\downarrow}\hat{b}_{l\downarrow} \\
&+ \sum_{ijkl}\sum_{\sigma\sigma'}\left(\sum_{\mu,\nu,\mu\neq\nu}\frac{V_{\mu\nu}}{2}c_{\mu,i}c_{\mu,j}c_{\nu,k}c_{\nu,l}\right)\hat{b}^{\dagger}_{i\sigma}\hat{b}_{j\sigma}\hat{b}^{\dagger}_{k\sigma'}\hat{b}_{l\sigma'} \\
&- \sum_{ij}\sum_{\sigma}\left[\sum_{\mu,\nu,\mu\neq\nu}\frac{V_{\mu\nu}}{2}(Z_{\nu}c_{\mu,i}c_{\mu,j}+Z_{\mu}c_{\nu,i}c_{\nu,j})\right]\hat{b}^{\dagger}_{i\sigma}\hat{b}_{j\sigma}
\end{aligned} \tag{3}$$

(symbols defined in the main text, Section 2). The above Hamiltonian is written on the basis defined by the ground state configuration and the configurations obtained by accounting for a single electron moved from the occupied MO to the virtual MO (i.e., single CI case) or also accounting for double, triple, etc. excitations. Exact diagonalization (Lanczos algorithm) of the Hamiltonian in the selected basis returns the correlated eigenstates.

S2 Playing around with model parameters

Fig. 1S compares the absorption spectra calculated for 2T-N and 2T-7N accounting for the same (black curves) or different t values for C-C and C-N bonds (red curves). For 2T-7N, we also set $U_N^{aza} = U_N^{py} = 15$ eV.[2] For both molecules, we observe a slight blue-shift of all excitations when different t values are used vs the results obtained with a single t value, as in the main text. The ST gap is not significantly affected by the choice of the t-hopping: for 2T-N it goes from $\Delta E_{ST} = -0.19$ eV to -0.18 eV, when going for a single t to two different t values, while for 2T-7N, it goes from $\Delta E_{ST} = +0.01$ eV to $+0.06$ eV.

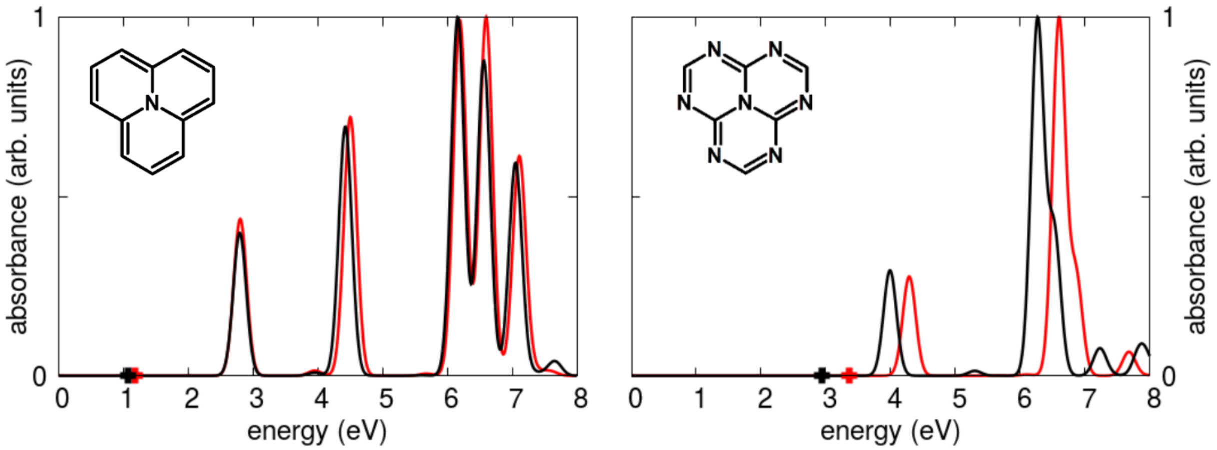

Figure 1S: Absorption spectra calculated for 2T-N and 2T-7N, with the same model parametyers as in the main text (black curves) and setting $t_{CN} = -2.5$ eV (red curves). For 2T-7N we also set $U_N^{aza} = U_N^{py} = 15$ eV. Crosses mark the position of the first excited (dark) state.

Fig. 2S compares 2T-N and 2T-7N absorption spectra as obtained in the main text (black curves, all bond lengths set to 1.4 Å) with those obtained for shorter (1.3 Å, green curves) and longer (1.5 Å, blue curves) bond lengths, and for the molecular geometries optimized at the B97-3c level from Ref.[3] (red curves). Relevant ΔE_{ST} values are reported in Tab. 1.

Fig. 3S shows results for 2T-7N obtained for different energy on the peripheral nitrogens, ε_N^{aza}. In the upper panel, an increasing blue-shift of the absorption bands and of first dark excited state is observed for more negative ε_N^{aza}. ΔE_{ST} goes from negative to positive values, moving from -0.09 eV for $\varepsilon_N^{aza} = -4.2$ eV to $+0.11$ eV for $\varepsilon_N^{aza} = -6$ eV. With reference to

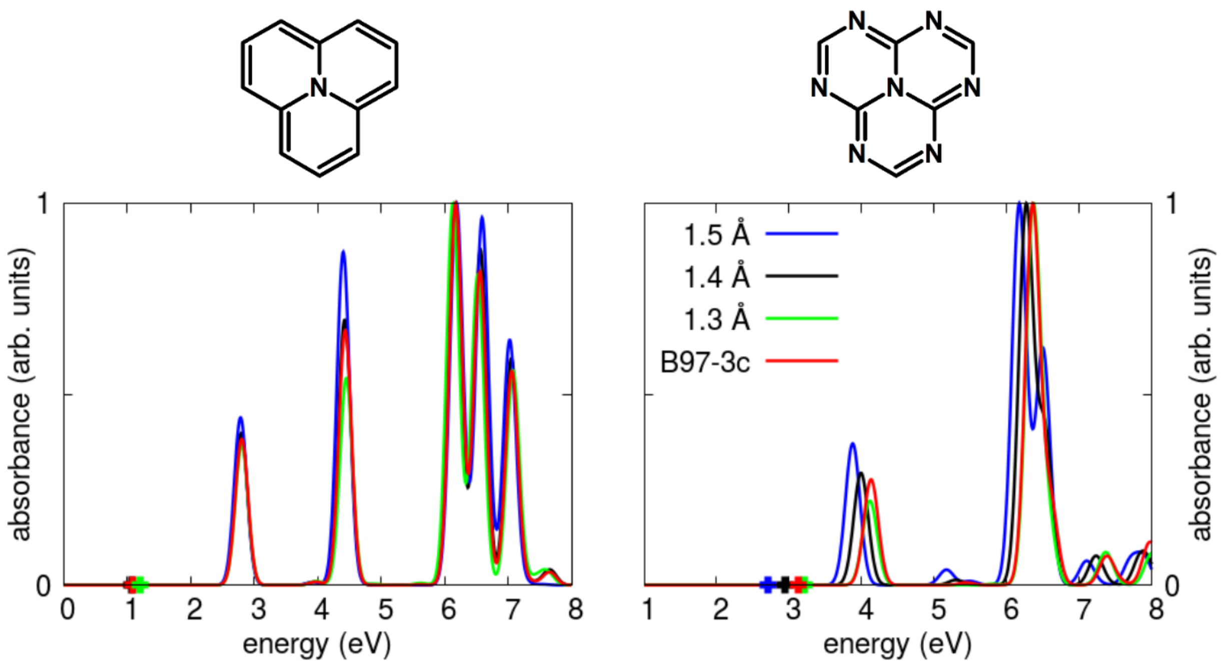

Figure 2S: Absorption spectra calculated by putting all bond lengths equal to 1.5 Å (blue curve), 1.3 Å (green curve) and for the optimized geometries at the B97-3c level from Ref.[3] (red curves) for 2T-N (left panel) and 2T-7N (right panel). Colored crosses are used to mark the position of the first excited dark state. Relevant results from the main text obtained by setting all bond lengths equal to 1.4 Å are reported in black. Same other model parameters used in Fig. 4 in the main text.

the experimental absorption spectrum (red curve in the upper panel), a good compromise is obtained for $\varepsilon_N^{aza} = -5$ eV (relevant results are shown as black bold lines).

Table 1: Singlet-triplet gap (eV units) for 2T-N and 2T-7N molecules obtained for different molecular geometries.

bond length	2T-N	2T-7N
1.5 Å	-0.22	-0.09
1.4 Å	-0.19	+0.01
1.3 Å	-0.15	+0.12
B97-3c	-0.18	+0.001

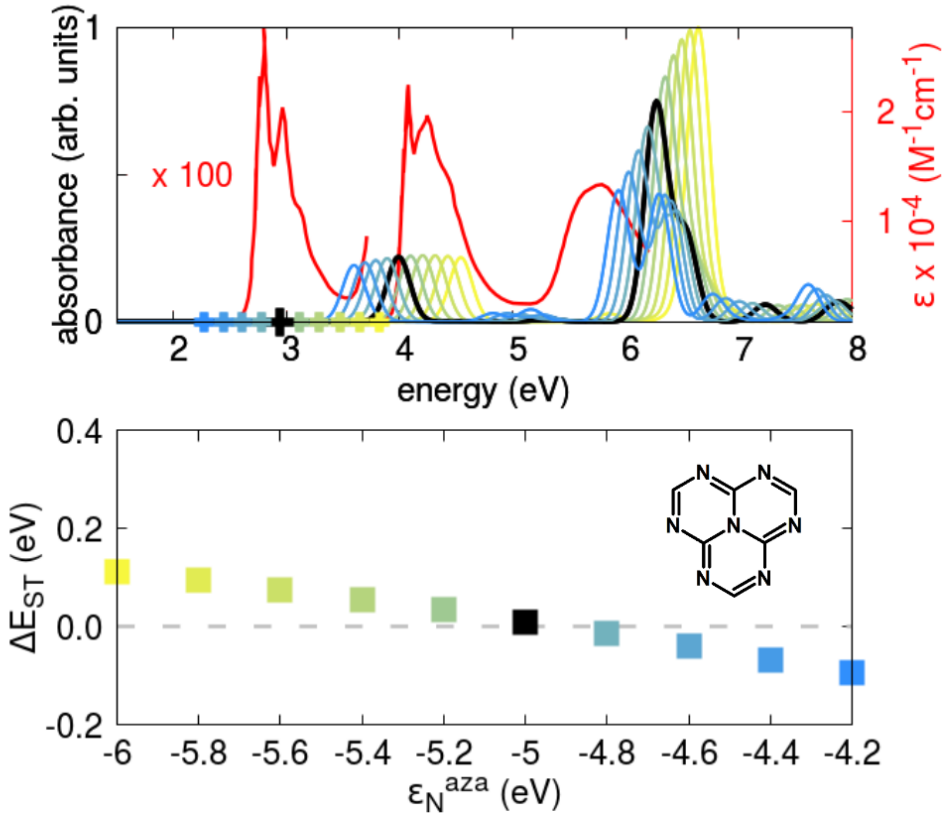

Figure 3S: Changing ε_N^{aza} in 2T-7N. Upper panel: Absorption spectra of 2T-7N: red curves are experimental data from Ref.,[4] colored lines show results for several ε_N^{aza} values (same color code used in the lower panel). Colored crosses in the low energy region are used to mark the position of the dark first excited singlet state. Calculated spectra are normalized to the maximum absorbance of the most intense spectrum. Lower panel: 2T-7N singlet-triplet energy gaps calculated for different ε_N^{aza} values. In both panels, black color is used to highlight results for $\varepsilon_N^{aza} = -5$ eV as relevant to the value used in the main text.

S3 Role of virtual double occupancy of the HOMO

In Fig. 4S a and b, we show the relative weight of the Hartree-Fock ground state together with the weights of the first few CIs of 2T-N and 2T-7N. As can be seen, the double occupancy of the HOMO (black curve) plays an important role only in the ground state, being completely absent in the excited states.

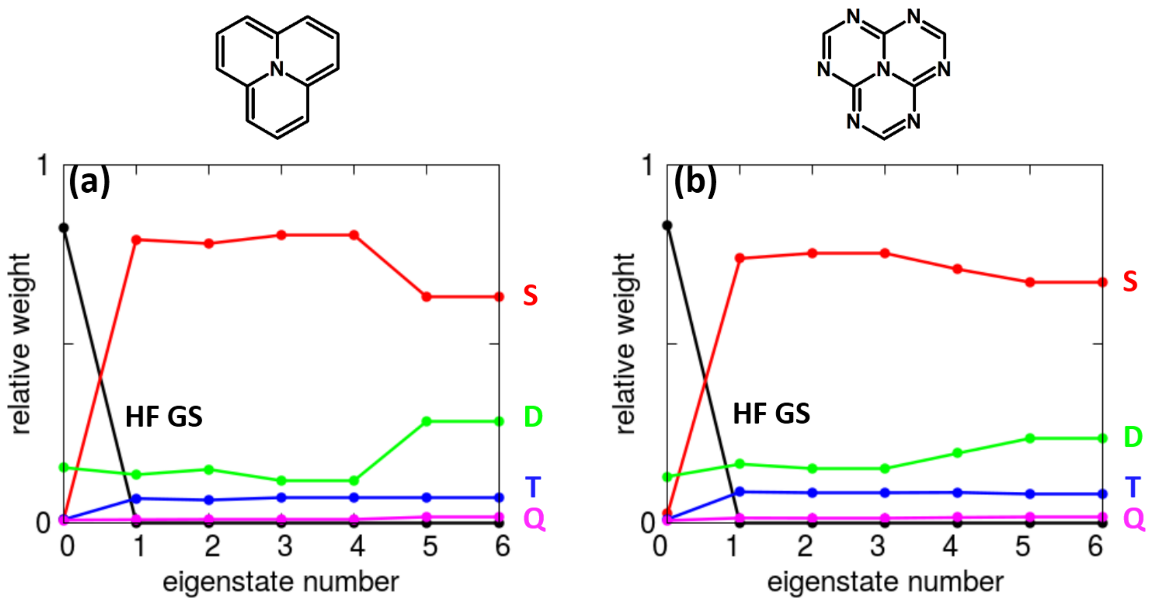

Figure 4S: Relative weight of the Hartree-Fock ground state (HF GS) together with the relative weights of single (S), double (D), triple (T), quadruple (Q) excitations for the first seven singlet and triplet eigenstates of 2T-N (panel a) and 2T-7N (panel b) calculated at the CISDTQ level. Model parameters defined in the main text.

S4 Ab initio CIS(D) results for 2T-N and 2T-7N

Ab initio CIS(D) calculations are performed with the Orca package (version 5.0.3), using the def2-TZVP basis set together with the auxiliary basis set def2-TZVP/C, after geometry optimization with the B97-3c method. In Tab. 2, we show the corrected energies together with their initial values for the first few excited states of 2T-N and 2T-7N. Very large corrections are calculated for singlet states, ranging from $\sim$ 1 eV for 2T-N to 1.8 eV for 2T-7N. Smaller corrections apply to triplet states.

S5 The effect of central connectivity on optical spectra

Fig. 5S shows absorption spectra calculated for systems with an increasing number of broken bonds between the central pyrrole nitrogen in 2T-N and 2T-7N to the molecular rim. When the molecular symmetry deviates from D_{3h} (see panels b and c), the low-lying excited states

Table 2: Ab initio CIS(D) results for 2T-N and 2T-7N. First few excited singlet and triplet states. All values are in eV.

state	2T-N corrected energy, E_c	2T-N initial energy, E_i	2T-N $E_i - E_c$	2T-7N corrected energy, E_c	2T-7N initial energy, E_i	2T-7N $E_i - E_c$
$^1A_2'$	1.050	1.802	0.752	2.650	4.339	1.689
$^3A_2'$	1.328	1.464	0.136	3.167	3.928	0.762
$^1E'$	3.103	4.224	1.120	4.432	6.302	1.870
$^3E'$	2.435	2.505	0.071	3.963	4.474	0.511

gain sizeable oscillator strength.

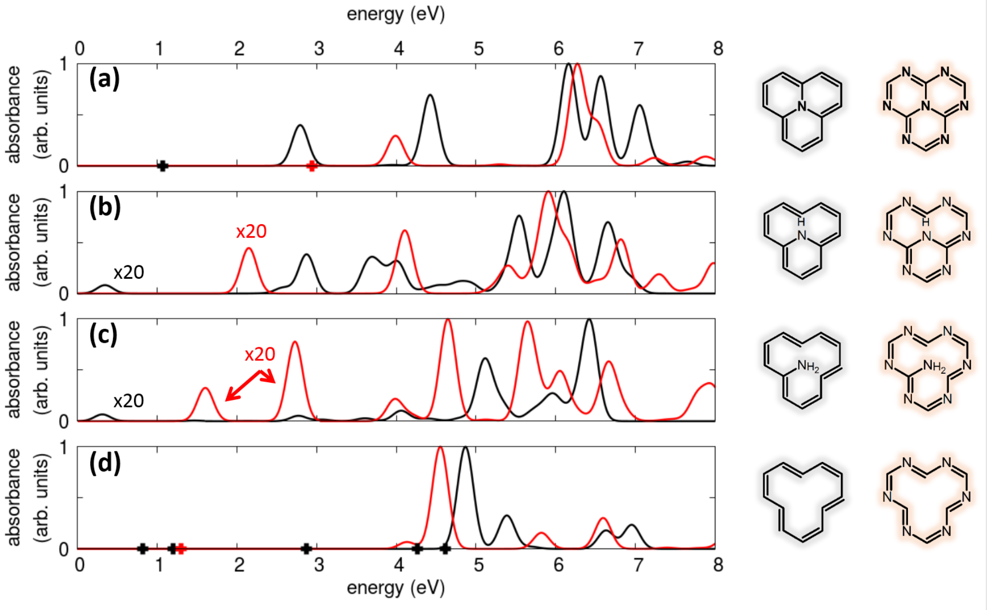

Figure 5S: Absorption spectra calculated (PPP CISDT level, model parameters as in Fig. 4, main text) for systems with an increasing number of broken bonds between the central nitrogen and the rim. Black and red curves and crosses refer to 2T-N and 2T-7N, respectively.

S6 Additional results for $(CHN)_x$ rings

In Figure 6S, we report the energy levels calculated at the Hückel and Hartree-Fock theory levels for the systems discussed in Fig. 8, main text, together with their carbon-only counterparts.

Figure 7S reports ΔE_{ST} values from Fig. 8, main text, together with the relevant results

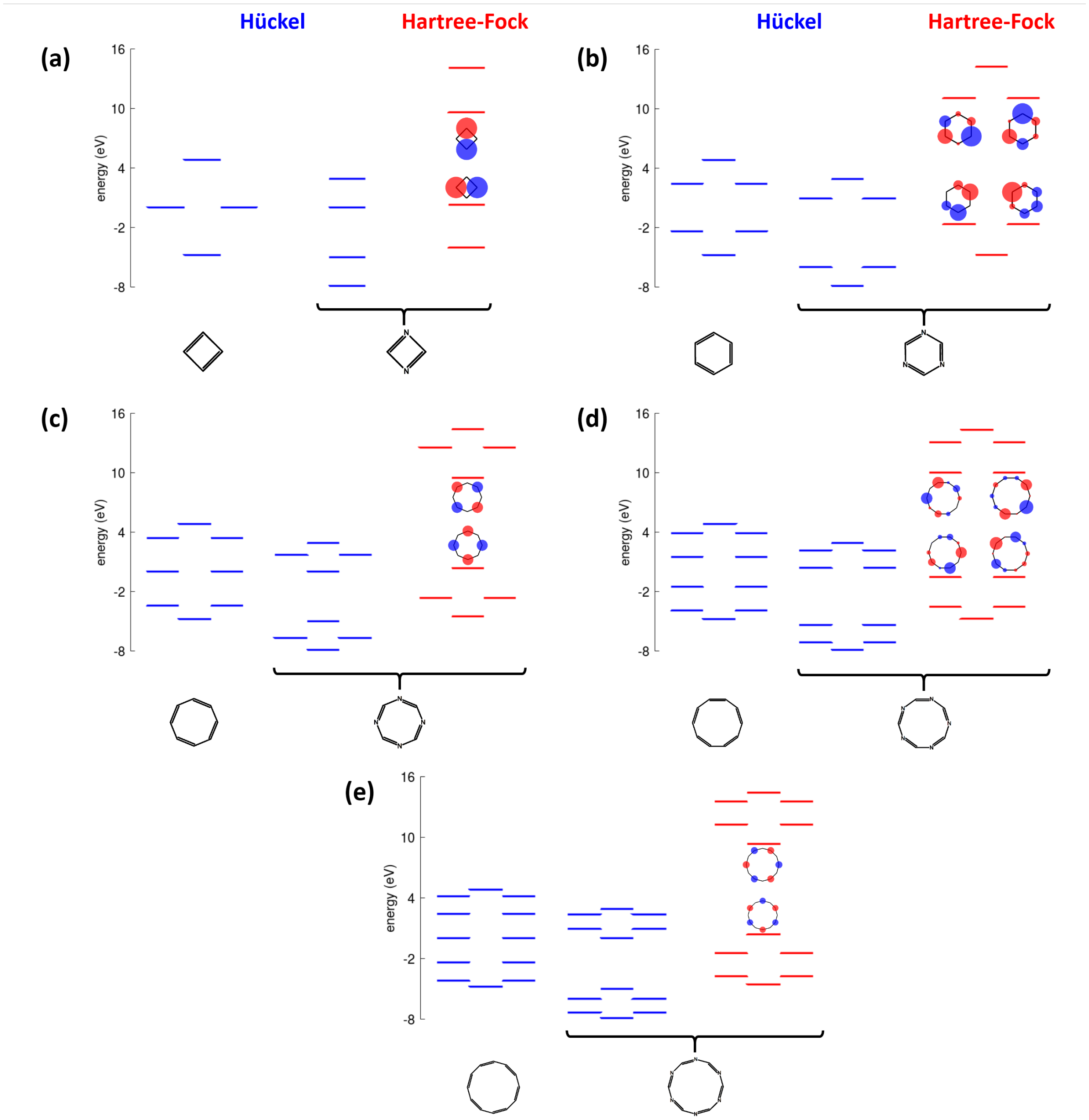

Figure 6S: Energy levels calculated at the Hückel (blue lines) and HF (red lines) theory levels for the rings studied in Fig. 8, main text, together with their carbon-only counterparts. Frontier HF-MOs are also shown for $(CHN)_x$ rings.

obtained at the PPP-CISD theory level.

S7 Validation against high-quality *ab initio* results

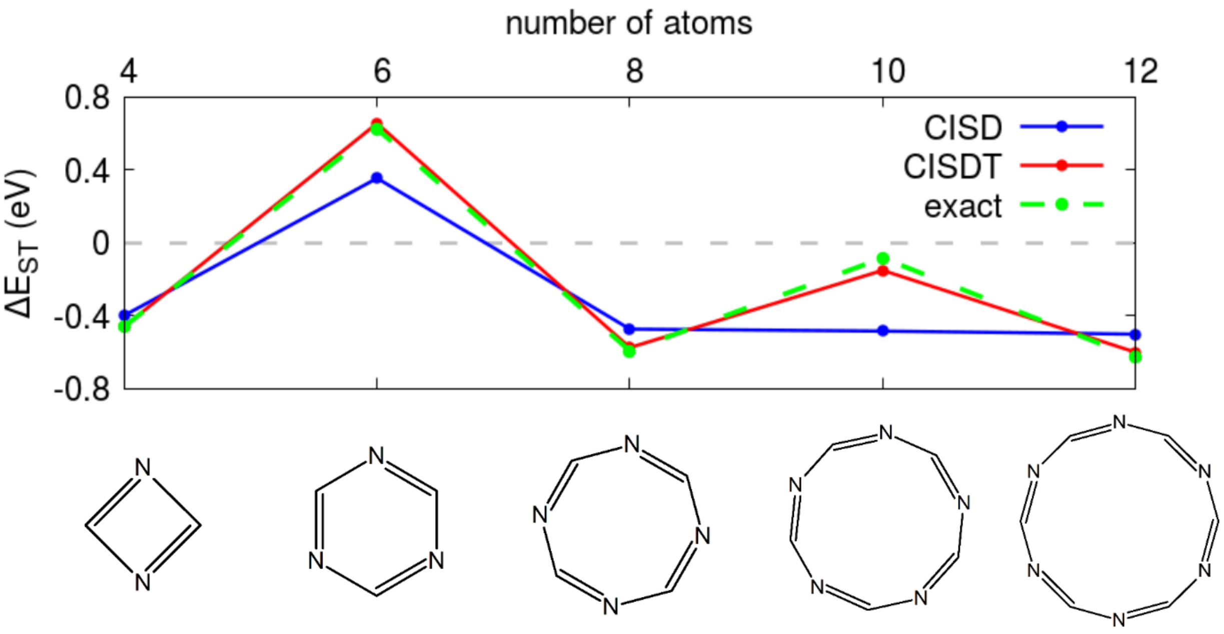

Figure 7S: ST gap values for rings with different numbers of aza nitrogen atoms. The blue curve refers to PPP CISD results, while the red curve to PPP CISDT results. Dashed green line refers to results from exact diagonalization in the RS basis (full-CI). Same model parameters used in Fig. 8 in the main text.

In Table 3, we report ΔE_{ST} values calculated at the Coupled Cluster (CC2 and CC3) theory level for the two smallest $(CHN)_x$ rings, namely $(CHN)_2$ and $(CHN)_3$, using Dalton program.[5,6] The calculations on these small molecules are computationally demanding. Crucially, CC3 calculations for the first eight excited singlet and triplet states took $\sim$ 30 hours on 64 cores for $(CHN)_2$ and $\sim$ 60 hours on 64 cores for $(CHN)_3$ on Intel Xeon E5-5218 2.3 GHz. Such timescales make it clear that it is impractical considering larger systems at this level of theory. Results for $(CHN)_3$ are obtained for the equilibrium geometry, as reported in Ref.[7] This geometry is very close to the idealized geometry adopted in the PPP analysis, so that results are directly comparable. On the other hand, in the case of $(CHN)_2$, equilibrium geometry (in Table 4) largely deviates from the idealized geometry adopted for PPP calculation, therefore CC2 and CC3 results are reported for both geometries. We notice that forcing the molecule in the idealized geometry leads to negative transition energies, an artefact that is cured in the equilibrated geometry. In both geometries, however, a negative ST gap is

observed, even if reduced in the equilibrium geometry vs the idealized geometry. Extending the basis from 6-31G* to 6-31G*+ marginally affects the calculated gaps. For $(CHN)_3$ the ST gap calculated with both CC2 and CC3 is positive and of similar magnitude, while, for $(CHN)_2$ they are negative, CC3 results being, in absolute value, considerably smaller than CC2 results.

Coupled Cluster and PPP-CISDT results are well in line (see Fig. 7S), with $(CHN)_2$ showing ST inversion and $(CHN)_3$ featuring a positive ST gap. A quantitative agreement is definitely not expected in view of the many approximations introduced in the PPP approach.

Table 3: Coupled Cluster ΔE_{ST} values for $(CHN)_2$ and $(CHN)_3$ as relevant to the two lowest $\pi \rightarrow \pi^*$ singlet and triplet transitions. Results are reported for two different basis sets. All values are in eV.

	$(CHN)_2$	$(CHN)_2^{eq}$	$(CHN)_3$
CC2/6-31G*	-0.49	-0.19	0.89
CC2/6-31G*+	-0.47	-0.19	0.85
CC3/6-31G*	-0.29	-0.1	1.0
CC3/6-31G*+	-0.28	-0.08	0.96

Table 4: Optimized cartesian coordinates of $(CHN)_2^{eq}$ at the DFT level (M06-2X functional, using cc-pVDZ basis set). All values are in Å.

atom type	x	y	z
C	0.00022	0.78045	-0.07934
C	-0.00022	-0.78045	-0.07934
H	-0.00035	-1.65975	-0.73666
H	0.00036	1.65975	-0.73666
N	1.10419	-0.00021	0.17324
N	-1.10419	0.00021	0.17324

S8 The HOMO-LUMO exchange energy

The HOMO-LUMO exchange integral reads:

$$K_{H-L} = \frac{e^2}{4\pi\varepsilon_0} \left\langle \psi_H(1)\psi_L(2) \left| \frac{1}{r_{12}} \right| \psi_L(1)\psi_H(2) \right\rangle \tag{4}$$

By expanding the MOs on the AO basis and adopting the ZDO approximation, Eq. 5 becomes:

$$K_{H-L} = \sum_{\mu\nu} c_{H\nu} c_{L\nu} c_{L\mu} c_{H\mu} V_{\nu\mu} \tag{5}$$

where the double sum runs over the AOs, $c_{H\nu}$ is the coefficient of ν AO on the HOMO ($c_{L\nu}$ is the same for LUMO) and $V_{\nu\mu}$ is the electrostatic repulsion between electrons on sites μ and ν, as introduced in Eq. 1 (main text). Looking at Eq. 5, it is clear that the exchange energy vanishes for disjoint HOMO and LUMO orbitals, but it may stay small in other cases as well. The HOMO-LUMO exchange energy is negligible in the aza-doped rings $(CHN)_{x=2,4,6}$, while it is not well-defined in $(CHN)_{x=3,5}$ because of the HOMO and LUMO double degeneracy (see Fig. 6S). An easy-to-use (PPP-based) computational tool is made publicly available for the calculation of K_{H-L} (https://github.com/francescodimaiolo/Hartree-Fock_PPP_tool).

References

(1) Mukhopadhyay, S.; Topham, B. J.; Soos, Z. G.; Ramasesha, S. Neutral and Charged Excited States in Polar Organic Films: Origin of Unusual Electroluminescence in Tri-*p*-tolylamine-Based Hole Conductors. *The Journal of Physical Chemistry A* **2008**, *112*, 7271–7279.

(2) Thomas, S.; Pati, Y.; Ramasesha, S. Linear and nonlinear optical properties of expanded porphyrins: A DMRG study. *The Journal of Physical Chemistry A* **2013**, *117*, 7804–7809.

(3) Ricci, G.; San-Fabián, E.; Olivier, Y.; Sancho-García, J. C. Singlet-Triplet Excited-State Inversion in Heptazine and Related Molecules: Assessment of TD-DFT and *ab initio* Methods. *ChemPhysChem* **2021**, *22*, 553–560.

(4) Halpern, A. M.; Rossman, M. A.; Hosmane, R. S.; Leonard, N. J. Photophysics of the $S_1 \longleftrightarrow S_0$ transition in tri-s-triazine. *The Journal of Physical Chemistry* **1984**, *88*, 4324–4326.

(5) Aidas, K. et al. The Dalton quantum chemistry program system. *WIREs Computational Molecular Science* **2014**, *4*, 269–284.

(6) Dalton, a molecular electronic structure program, Release v2020.1 (2022), see http://daltonprogram.org.

(7) Loos, P.-F.; Lipparini, F.; Boggio-Pasqua, M.; Scemama, A.; Jacquemin, D. A Mountaineering Strategy to Excited States: Highly Accurate Energies and Benchmarks for Medium Sized Molecules. *Journal of Chemical Theory and Computation* **2020**, *16*, 1711–1741.
